# Administration of DDAVP did not improve the pharmacokinetics of FVIII concentrate in a clinically significant manner

**Published:** 2018-02-21

**Authors:** Janneke I Loomans, Eva Stokhuijzen, Marjolein Peters, Karin Fijnvandraat

**Affiliations:** ^1^Emma Children’s Hospital, Department of Pediatric Hematology, Academic Medical Center, University of Amsterdam, Amsterdam, the Netherlands; ^2^Department of Plasma Proteins, Sanquin Research, Amsterdam, the Netherlands

**Keywords:** Hemophilia A, factor VIII concentrate, von Willebrand Factor, half-life, desmopressin

## Abstract

**Background:**

The half-life and mean residence time (MRT) of infused recombinant factor VIII (FVIII) concentrate are associated with pre-infusion levels of von Willebrand factor (VWF) in severely affected hemophilia A patients. It is currently unknown if individual FVIII concentrate half-life and MRT can be extended by increasing endogenous VWF levels. **Aim: **Our aim was to evaluate the effect of a 1-deamino-8-D-arginine vasopressin (DDAVP)-induced rise in VWF concentration on the pharmacokinetics of infused FVIII in hemophilia A patients.

**Methods:**

Four adult hemophilia A patients participated in this cross-over, placebo-controlled study. Each patient received either intravenous DDAVP or placebo, one hour prior to administration of 50 IU/kg plasma-derived immune-affinity purified FVIII concentrate.

**Results:**

The combined administration of DDAVP and FVIII concentrate was well tolerated. The levels of VWF Antigen (Ag) doubled after DDAVP, whereas they remained stable after placebo infusion. This rise in VWF Ag resulted in a slight modification of the pharmacokinetic parameters of FVIII concentrate. The MRT of FVIII concentrate increased in all patients (mean from 17.6 h to 19.9 h, p < 0.001, 95% CI for MRT change: +4.7 to -0.3 h). However, in vivo recoveries tended to decrease following DDAVP administration.

**Conclusions:**

Collectively, these data show that administration of DDAVP did not improve the pharmacokinetics of FVIII concentrate in a clinically significant manner.

**Relevance for patients:**

Our results indicate that no clinical benefit is to be expected from the modification in FVIII pharmacokinetics resulting from DDAVP-administration prior to infusion of FVIII concentrate in hemophilia A patients.

## 1. Introduction

Hemophilia A is a congenital bleeding disorder caused by a deficiency of blood coagulation factor VIII (FVIII)[1]. The treatment of severely affected patients consists of intravenous administration of FVIII concentrate, either as prophylaxis or when bleeding occurs. To maintain adequate levels of FVIII after major bleeding or surgery, or in prophylactic treatment regimens, frequent administration of FVIII concentrate is necessary, since the half-life of infused FVIII is approximately 8-12h [ 2 ]. FVIII is bound non-covalently to von Willebrand factor (VWF) in plasma and is thereby protected from proteolytic degradation [3].

**Figure 1.**
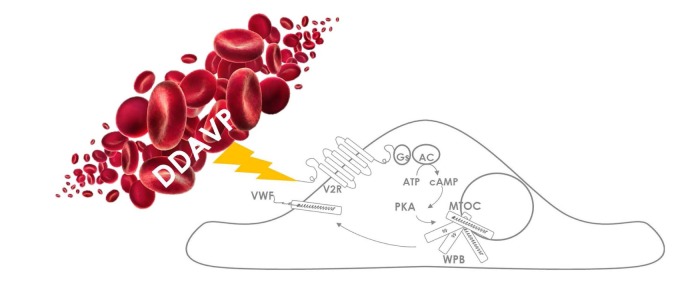
Endothelial cell release of VWF upon stimulation with DDAVP [5,8]. VWF is packaged into elongated secretory storage organelles designated Weibel-Palade bodies (WPBs) in endothelial cells. Upon DDAVP stimulation, WPBs release VWF indirectly by acting on vasopressin 2 receptors (V2R) that are expressed on lung endothelial cells [9]. Binding of DDAVP to V2R activates a protein kinase (PKA) dependent signaling pathway that induces release of WPBs via Gs [5,8,9]. Gs is an alpha subunit of a receptor linked hetero-trimeric G protein that activates adenylate cyclase (AC) that catalyzes the formation of cAMP. In response to cyclic adenosine monophosphate (cAMP) raising agonist a subset of WPBs has been shown to cluster at the microtubule organizing centre (MTOC) which is located close to the nucleus. Subsequently, WPBs travel to the cell membrane and release VWF.

VWF is a multimeric glycoprotein synthesized by vascular endothelial cells and megakaryocytes [4]. After synthesis in endothelial cells, it is either directly released in the plasma or packaged into elongated secretory storage organelles designated Weibel-Palade bodies (WPBs) [5,6]. The stored VWF can be released upon administration with the synthetic vasopressin analogue, 1-deamino-8-D-arginine vasopressin (DDAVP). DDAVP is the synthetic analogue of the pituitary hormone vasopressine that is released following adrenergic stimulation in stressful situations [ 7 ]. Figure 1 shows the mechanism of action of DDAVP.

In general, the survival of FVIII following intravenous administration is dependent on circulating VWF. The importance of VWF for sustained levels of circulating FVIII is clearly demonstrated in patients with the severe type of von Willebrand disease (vWD), in whom no circulating VWF is detectable. Morfini et al. showed that the half-life of a plasma- derived FVIII concentrate is strongly reduced in 11 vWD patients (mean 2.8 h) in comparison to hemophilia A patients (mean 10.5 h), who have normal levels of VWF [10]. The short half-life of FVIII in severe vWD patients could be prolonged by increasing VWF-levels prior to administration of FVIII concentrate through administration of cryoprecipitate [11].

Although the half-life of FVIII concentrate is about 8-12 h in hemophilia A patients, large variability between individuals (6-24 h) has been observed [2,12,13]. Much of the variation is related to the clearance of VWF, which is dependent on VWF plasma levels, blood group, and age [3,13-15]. FVIII concentrate half-life increases with age, whereas it shortens during active bleeding, after surgery or during infection [16]. The variation in FVIII concentrate half-life in hemophilia patients is strongly associated with pre-infusion VWF-levels [13]. An increase of 0.1 IU/mL in VWF antigen (Ag) level results in a 16.6 min prolongation of FVIII concentrate half¬life in severe hemophilia A patients [17].

It is currently unknown why levels of VWF Ag are associated with FVIII concentrate half-life. Barnes et al. proposed two potential hypotheses to explain this observation [18]. The first hypothesis states that the heterogeneity of VWF Ag is caused by different rates of VWF clearance. Clearance of VWF and FVIII occurs most often as a complex in the liver and spleen[4]. According to this hypothesis, low VWF Ag levels would be a result of an increased clearance rate of both VWF and FVIII, which may result in a decreased FVIII concentrate half-life [18].

The second hypothesis Barnes et al. described entails the capacity of the concentration of VWF itself to affect the pharmacokinetics of FVIII concentrate.

These observations and hypotheses raise the question whether it is possible to prolong FVIII concentrate half-life in hemophilia A patients by increasing their pre-infusion endogenous VWF-levels. This rise can be achieved by administration of DDAVP prior to the administration of FVIII concentrate. VWF released from WPBs upon DDAVP stimulation is known to contain large amounts of high molecular weight VWF multimers, which possess the greatest hemostatic capacity [6]. Furthermore, DDAVP exerts its hemostatic effect also by the enhancement of platelet coagulant activity, but the exact mechanism of action remains to be elucidated [19].

Deitcher et al. conducted a study to test whether administration of a 300-^g intranasal dose of DDAVP to ten hemophlia A patients would improve pharmacokinetic properties of high-purity and recombinant FVIII concentrates [20]. They observed a modest increase in VWF Ag levels upon DDAVP stimulation without a significant effect on FVIII concentrate half-life or clearance. Their conclusion states however that any modest increase of FVIII concentrate half-life upon artificially induced VWF levels may alter the pharmacokinetics of infused FVIII concentrates. The aim of this study is to evaluate the effect of a DDAVP-induced rise in VWF concentration by intravenous administration on the pharmacokinetics of infused FVIII in hemophilia A patients.

## 2. Materials and Methods

### 2.1 Study design

This randomized, double blind cross-over study was approved by the Institutional Review Board of the Academic Medical Center in Amsterdam. Each patient received two administrations of 50 IU/kg FVIII concentrate, with a time- interval of at least two weeks. The FVIII administration was preceded by intravenous administration within 30 minutes of either 0.3 gg/kg DDAVP, dissolved in 100 ml NaCl 0.9%, or 100 ml NaCl 0.9% (placebo). FVIII concentrate was administered one hour after the end of the DDAVP or placebo infusion, since intravenous DDAVP achieves a maximal effect after one hour. Randomization and double blind preparation of the DDAVP solution was performed at the pharmacy of the Academic Medical Center according to good clinical and good manufacturing practices (GCP and GMP).

The study was performed in accordance with the International Society on Thrombosis and Hemostasis (ISTH) guidelines for pharmacokinetic studies for FVIII concentrates [21]. A wash-out period of at least four days was observed for previously administered FVIII concentrate. At ten different time points, up to 48 hours after the FVIII administration, blood was collected in tubes, containing 10% volume/volume final concentration sodium citrate 3.2%. The blood samples were centrifuged for ten minutes at 4000 g at ambient room temperature and plasma aliquots were stored at -70 degrees Celsius.

Prior to the administration of DDAVP (or placebo) and one hour after the administration of FVIII concentrate, a blood sample was also collected in EDTA for platelet counting and for measurement of hemoglobin and hematocrit, using a Coulter Counter STKS or STKR. One hour after DDAVP or placebo administration and ten minutes after FVIII concentrate administration blood pressure and pulse rate were recorded.

### 2.2 FVIII concentrate preparation

The FVIII concentrate used was a plasma-derived immuno- affinity purified concentrate (CLB-Factor-VIII-M), manufactured by Sanquin, Amsterdam [21]. After reconstitution, this solvent detergent (SD) treated FVIII concentrate contains 100 IU/ml (40nM) FVIII and (50 nM) VWF, as derived from the product specification. The molar ratio of FVIII and VWF is 1:1 in the preparation used.

The study was performed with one lot of FVIII concentrate.

### 2.3 Analysis of plasma concentrations of FVIII, VWF and VWF-propeptide

Plasma FVIII concentration was assessed by a chromogenic substrate assay using a plasma standard calibrated against the third International Standard (WHO 91/666) for FVIII. FVIII Ag, VWF Ag and VWF- propeptide Ag were measured as described previously [23,24]. To determine VWF ristocetin cofactor activity, platelet aggregation was measured in a platelet activation profiler (Bio/Data Corporation, Hatboro, PA, USA) after stimulation with ristocetin (AggRecetin, Bio/Data Corporation, Hatboro, PA, USA). VWF multimeric structure was analyzed by 2.5% SDS agarose gel electrophoresis.

### 2.4 Pharmacokinetic analysis

Plasma FVIII concentration prior to and at 10 and 30 minutes and 1, 3, 6, 10, 24, 32 and 48 hours after FVIII administration were used to calculate pharmacokinetic parameters. If the FVIII concentration increased after DDAVP administration, we adjusted for the DDAVP-induced rise in FVIII.

Pharmacokinetic parameters were calculated by model independent methods [25]. The area under the curve (AUC; units: h/dl) was calculated from the concentration versus time curve and the area under the first moment curve (AUMC; units hours/dl) was calculated from a curve of the product of concentration and time versus time. Both areas were calculated using the trapezoidal rule. The conventional elimination half¬life was calculated from the regression line of the FVIII concentration versus time curve. The half-life of the released VWF propeptide was calculated by a one compartment model.

Statistical comparisons were performed by paired Student's t-test.

## 3. Results

### 3.1 Patients

Four hemophilia A patients were enrolled in this study after informed consent was given ( Table 1). None of the patients had an inhibitor to FVIII (defined as >0.5 BU/ml), either at the time of the study or in the patient's history. At the time of the study, the patients did not have active bleeding and were not treated with FVIII concentrate for at least four days, prior to the start of the study. All patients were HIV seronegative and hepatitis C seropositive. There were no signs or symptoms of liver failure and the patients did not use alpha-interferon. None of the patients was affected by concomitant cardiac or renal disease.

### 3.2 Clinical study

The combination of DDAVP and FVIII concentrate was well tolerated. The concentration of VWF showed a significant increase in all four patients one hour after DDAVP administration, from a mean value of 1.35 (range 1.2-1.7) to 2.69 (range 2.2-3.1) IU/ml.

As expected, there was an increase in high multimeric forms of VWF after DDAVP infusion, which did not occur after the infusion of placebo (data not shown). The concentration of the VWF-propeptide in plasma, serving as a marker of DDAVP-induced VWF release, showed a simultaneous 5-fold increase (mean: 6.2 (range 3.9-8.8) nM to 28.9 (range 24.3-35.4) nM) [24]. The half-life of the released VWF- propeptide was 3.4 h (range: 3.2-3.5 h). The placebo did not influence the VWF Ag levels.

### 3.3 Pharmacokinetics

The DDAVP-induced rise in VWF resulted in a slight modification of the pharmacokinetic parameters of FVIII  concentrate (Table 2).

**Figure 2.**
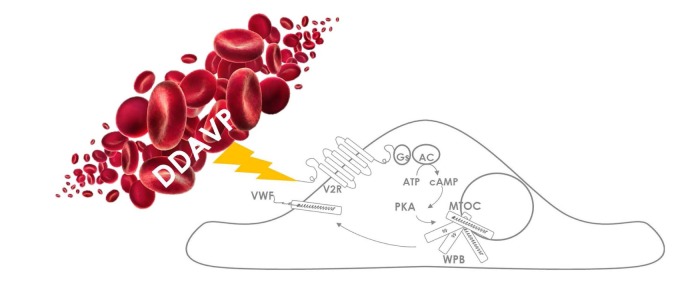
Elimination curves of VWF (**A**) and FVIII concentrate (**B**) in all patients. **(A) **Concentration of VWF Ag over time, after administration of placebo and FVIII concentrate (blue line) or DDAVP and FVIII concentrate (orange line) **(B) **FVIII concentrate elimination curves of the same patients after infusion of FVIII concentrate preceded by placebo or DDAVP administration. All elimination curves are adjusted for baseline and DDAVP induced FVIII levels.

In all patients, the MRT of FVIII concentrate was prolonged after DDAVP administration compared to placebo, with a mean MRT increase from 17.6 to 19.9 h (p = 0.07, 95% C.I. for MRT change: +4.7 to -0.3 hr). After administration of DDAVP there was a tendency towards a lower recovery of FVIII concentrate activity and an increase in its reciprocal equivalent, the volume of distribution. These differences did not reach statistical significance. The elimination curves of both FVIII concentrate and VWF Ag for all patients are depicted in Figure 2. Although the MRT of FVIII concentrate increased, this did not result in higher FVIII concentrate activity level at the post-transfusion time-points for patient 1 (Figure 2B).

**Table 1.**
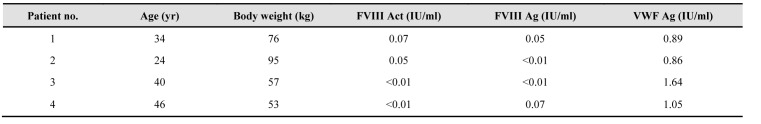
Four hemophilia A patients enrolled after informed consent

Patient characteristics. The FVIII concentration increased after DDAVP administration in patient no. 1 and no 2. In both patients we adjusted for the DDAVP induced rise in FVIII in the concentration time curve.

**Table 2.**
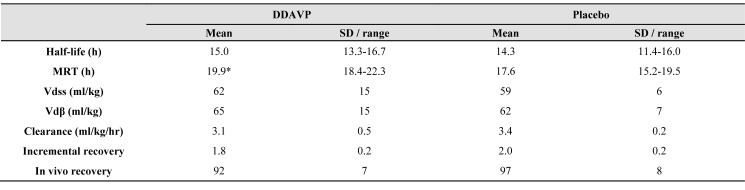
The DDAVP-induced rise in VWF resulted in a slight modification of the pharmacokinetic parameters of FVIII concentrate

Pharmacological and pharmacokinetic parameters of FVIII concentrate activity studied. MRT: mean residence time, Vdss: volume of distribution at steady state,Vdp: volume of distribution during elimination phase 95% C.I. for change of MRT after DDAVP administration: + 4.7 hr to - 0.3 hr 95% C.I. for change of half-life after DDAVP administration: + 4.8 hr to - 3.3 hr * p = 0.07.

## 4. Discussion

Previously, it was demonstrated that the FVIII concentrate half-life is associated with pre-infusion levels of VWF in severely affected hemophilia A patients [13]. The present study demonstrates that doubling pre-infusion VWF concentration by DDAVP results in a minor prolongation of the MRT of FVIII concentrate. Our results are more in agreement with a later study, demonstrating that nasal DDAVP administration prior to FVIII concentrate administration significantly increased both MRT and volume of distribution of FVIII concentrate [20].

Our observations suggest that doubling of the plasma concentration of VWF induced by DDAVP, does slightly increase FVIII concentrate survival. This is intriguing from a stoichiometric point of view, because VWF circulates in a 100-fold molar excess over FVIII [26]. However, not all the binding sites that are in principle available in the VWF multimers may be accessible. Moreover, since the kinetics of FVIII concentrate and VWF in plasma are governed by multiple binding and elimination equilibriums, the effect of changes in VWF concentration may influence FVIII concentrate pharmacokinetics.

A mathematical model was published that accurately predicted a wide range of pharmacokinetic observations of FVIII concentrate reported in the literature [27]. The clinical data concerning the association of FVIII concentrate half-life and the baseline plasma concentration of VWF in hemophiliacs fitted remarkably well to this model [13,27]. Based on this model, a more pronounced increase in FVIII concentrate half-life would be expected with the rise in VWF concentration observed in the present study. Apparently, this is not the case. We identified three possible explanations for the limited effect of a DDAVP induced rise of VWF on the pharmacokinetics of FVIII concentrate.

First, the potential increase in FVIII concentrate half-life by increased VWF-levels may be off-set by the short half-life of the high molecular weight multimers of VWF that are released following DDAVP [ 28,29 ]. The binding of FVIII is not influenced by the degree of multimerization of VWF, as is evident from the in vitro VWF binding experiments in this study (not shown) and data reported before [30,31]. When the proportion of highly multimerized VWF is increased after DDAVP administration, a higher fraction of the administered FVIII concentrate will bind to high molecular weight VWF multimers. However, the clearance of these multimers is enhanced compared to less multimerized forms of VWF [32]. Therefore, the clearance of FVIII concentrate bound to these multimers may also be enhanced, resulting in a net effect of a shorter half-life.

Second, the FVIII concentrate used in this study contains residual amounts of VWF which might partially obscure the effect of the DDAVP induced rise of VWF. It is difficult to estimate the effect of the residual amount of VWF in the product on our outcomes, as knowledge is scant on the exact interaction between VWF and FVIII. An early study on the binding of FVIII to VWF provided evidence for multiple classes of FVIII-binding sites on VWF [33]. However, this study concluded that “The high-affinity binding (Kd = 2.1 x 10(-10) M) was restricted to only 1-2% of the VWF subunits. Competition studies with monoclonal antibodies with known epitopes demonstrated that the FVIII sequence Lys1673- Arg1689 is involved in the high-affinity interaction with VWF.” This observation is consistent with the molar ratio of FVIII/VWF of 1:50 in the circulation. It is currently not known why only 1-2% of the VWF subunits can bind FVIII with high affinity. In the product used in this study, the molar ratio of FVIII/VWF was 1:1. Using velocity sedimentation analysis Lollar and Parker provided evidence for the presence showed that one factor VIII-binding site is present per VWF monomer, these results indicate that all factor VIII-binding sites are accessible in the VWF multimer [34].

The differences observed between the two studies show that the stoichiometry of binding may depend on the technology used to assess binding. We suspect (but we cannot prove) that the differences observed are due the presence of multiple classes of binding sites for FVIII on VWF. Low affinity binding of FVIII may occur to all VWF subunits yielding a stoichiometry of 1:1 whereas high affinity binding of FVIII may occur to a subset of VWF subunits (for instance the terminal VWF subunits in a VWF multimer) with a stoichiometry of binding of 1:50. Competition studies with monoclonal antibodies with known epitopes demonstrated that the FVIII sequence Lys1673-Arg1689 is involved in the high- affinity interaction with VWF [33]. Furthermore, sulfation of Tyr1680 has been shown to be a crucial for high affinity binding to VWF [35]. The physiological importance of this high affinity binding site on FVIII for VWF is suggested by the low FVIII levels observed in patients carrying a Tyr1680 to Phe substitution (median baseline FVIII:C in 20 patients in INSIGHT study was 5 IU/dL (IQR 2-31)) [36].

In light of the ambiguity regarding the VWF present in the FVIII product which may obscure the DDAVP induced effect, we looked into patient one (No. 1). For this patient we had data available from a DDAVP administration without placebo or FVIII concentrate. The VWF Ag peak following DDAVP only was 2.6 IU/ml, which was almost as high as the VWF Ag peak following FVIII concentrate after DDAVP administration (2.5 IU/ml at time point 0.5 in Figure 2A). This indicates that the amount of VWF in the exogenous product is probably relatively small.

Third, patients with lower baseline VWF levels may have an increased clearance of VWF. The increased clearance may also be present after a DDAVP induced rise in these patients [18].

Although the MRT of FVIII concentrate was slightly prolonged, this did not result in a clinical benefit in FVIII concentrate survival curves. This apparent contradiction may be explained by the lower recovery of FVIII concentrate observed in three of the four patients after DDAVP administration. Expansion of the plasma volume as a result of the antidiuretic effect of DDAVP may partly account for this reduced recovery. The reduced recovery limits the beneficial effect of an increase in MRT for clinical purposes.

Unfortunately, we have no data on the genotype of the four hemophilia A patients. This information might have been helpful to explain our observations, as for example mutations that cause a VWF binding defect may impair the prolongation of FVIII concentrate half-life. Moreover, this information may also have helped us to explain the differences in FVIII Act and FVIII Ag in our patients.

In conclusion, our results indicate that no clinical benefit is to be expected from the modification in FVIII concentrate pharmacokinetics by DDAVP-administration prior to infusion of FVIII concentrate.
